# High-speed odor sensing using miniaturized electronic nose

**DOI:** 10.1126/sciadv.adp1764

**Published:** 2024-11-06

**Authors:** Nik Dennler, Damien Drix, Tom P. A. Warner, Shavika Rastogi, Cecilia Della Casa, Tobias Ackels, Andreas T. Schaefer, André van Schaik, Michael Schmuker

**Affiliations:** ^1^ Biocomputation Group, University of Hertfordshire, Hatfield AL10 9AB, UK.; ^2^ International Centre for Neuromorphic Systems, Western Sydney University, Kingswood, 2747 NSW, Australia.; ^3^ Sensory Circuits and Neurotechnology Laboratory, Francis Crick Institute, London NW1 1AT, UK.; ^4^ Department of Neuroscience, Physiology and Pharmacology, University College London, London WC1E 6BT, UK.; ^5^ Sensory Dynamics and Behaviour Lab, Institute of Experimental Epileptology and Cognition Research (IEECR), University of Bonn Medical Center, 53127 Bonn, Germany.; ^6^ BioML Research Services, Berlin, Germany.

## Abstract

Animals have evolved to rapidly detect and recognize brief and intermittent encounters with odor packages, exhibiting recognition capabilities within milliseconds. Artificial olfaction has faced challenges in achieving comparable results—existing solutions are either slow; or bulky, expensive, and power-intensive—limiting applicability in real-world scenarios for mobile robotics. Here, we introduce a miniaturized high-speed electronic nose, characterized by high-bandwidth sensor readouts, tightly controlled sensing parameters, and powerful algorithms. The system is evaluated on a high-fidelity odor delivery benchmark. We showcase successful classification of tens-of-millisecond odor pulses and demonstrate temporal pattern encoding of stimuli switching with up to 60 hertz. Those timescales are unprecedented in miniaturized low-power settings and demonstrably exceed the performance observed in mice. It is now possible to match the temporal resolution of animal olfaction in robotic systems. This will allow for addressing challenges in environmental and industrial monitoring, security, neuroscience, and beyond.

## Teaser

Miniaturized high-speed electronic nose matches timescales of animal olfaction, unlocking potential for mobile robotics.

## INTRODUCTION

**Fig. 1.**
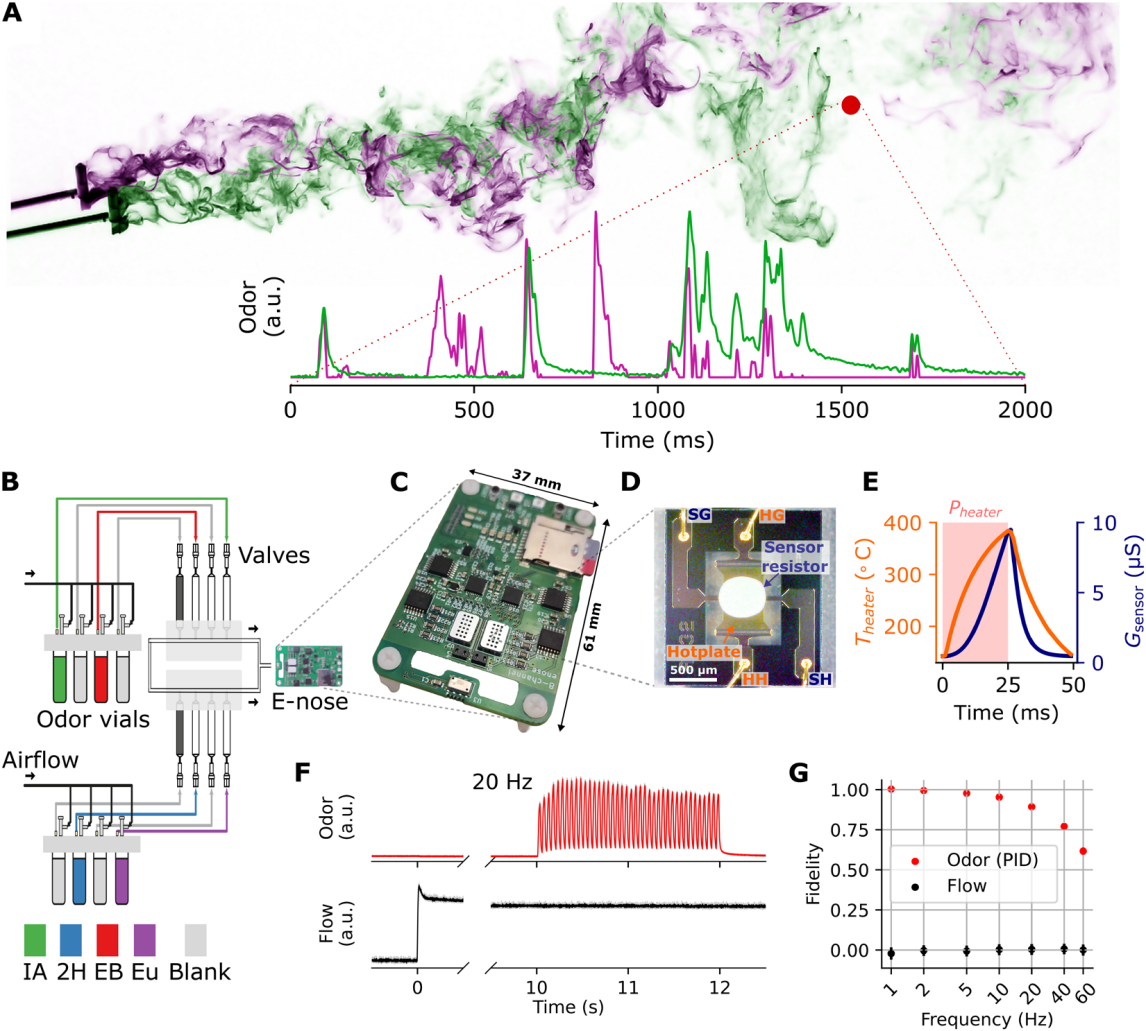
Electronic nose and odor delivery system. (**A**) Decoding temporal information of odor plumes requires fast sensing. Top: Two sequential TiCl_4_ smoke plume photographies, shifted and superimposed, provided by P. Szyszka. Bottom: Dual-PID recordings of source-separated odor plumes, from Ackels *et al.* (*24*). Plume and sensor location (red) for illustrative purposes only. (**B**) Experimental setup with odor delivery device and electronic nose. (**C**) Electronic nose circuitry. (**D**) Microscopy image of the MiCS-6814 NH3 sensor with its housing removed. (**E**) Heater modulation cycle in ambient air. (**F**) PID and flow meter traces for a 20-Hz stimulus. Solid/faded (occluded) traces for mean/SD. of five trials. (**G**) Resulting olfactometer temporal fidelity, for various frequencies. Odorants abbreviations: IA, isoamyl acetate; EB, ethyl butyrate; Eu, cineol; 2H, 2-heptanone; blank, odorless control.

The sense of olfaction is found all across the animal kingdom and is crucial for survival and guiding behaviors such as navigation, food detection, predator avoidance, and mate selection (*1*–*7*). Success in these tasks often hinges on the ability to swiftly and accurately detect and recognize scents (*8*–*11*), particularly when dealing with odor plumes characterized by brief and intermittent encounters (*12*, *13*) generated by turbulent dispersion processes (*14*–*16*). Concentration fluctuations in odor plumes can exceed 100 Hz (*17*), while individual odor encounters can last single milliseconds or less (*18*) (see Fig. 1A). Many environmental cues are embedded in the fine structure of the odor plume (*12*, *19*, *20*), which various organisms have evolved to use for their advantage. For instance, male locusts (*Schistocerca americana*) olfactory receptor neurons can transduce odors in less than 2 ms and resolve odor stimuli fluctuations at frequencies exceeding 100 Hz (*21*). Similarly, honeybee projection neurons decode odor identity in tens of milliseconds after stimulus onset (*22*), while mosquitoes can identify CO_2_ packets of just 30 ms (*23*). A recent landmark study in mice has revealed their ability to discriminate rapid odor fluctuations, enabling them to distinguish temporally correlated from anticorrelated odors at up to 40 Hz, which facilitates source separation in complex environments (*24*).

Research on mobile olfactory robotics (*25*) has flourished over the past decade, driven by promising applications and solutions across various domains (*26*) and bootstrapping on the well-established field of artificial olfaction (*27*). The latter has demonstrated its effectiveness in domains where static and slow measurements are sufficient, such as the detection of hazardous gases or pollutants (*28*), spoilage alert systems (*29*), health monitoring (*30*), and food sciences (*31*). However, many recent applications call for unmanned ground or aerial vehicles (UGV / UAV) to perform odor source localization and navigation tasks (*26*, *32*–*34*), which rely heavily on sensing the environment fast and efficiently, considering plume dynamics (*35*).

Typically, mobile olfactory robots incorporate electronic noses, devices that are characterized by arrays of multiple gas sensors and associated peripheral electronics (*36*). They offer distinct advantages over conventional analytical methods such as photoionization detectors (PIDs) and mass spectrometers, notably in terms of portability, power efficiency, cost-effectiveness, and sensitivity to a wide range of odors and volatile compounds.

The most widely used sensing components are metal-oxide (MOx) gas sensors (*37*), which offer the substantial advantage of a sensing layer that can be tuned through (i) modifications to its chemical structure and (ii) variations in operating temperature achieved by local heating, allowing for effectively detecting a diverse range of analyte classes.

Their minimal requirements for electronic peripheral components streamline sensor design, lower costs, and conserve valuable space. Further reductions in latency, form factor, and power consumption were enabled through latest micro-electromechanical systems (MEMS)–based MOx sensors (*38*, *39*), facilitating seamless integration into electronic circuits (*40*).

However, the relatively slow response and recovery times of MOx sensor electronic noses pose challenges for widespread adoption and are prohibitive for many potential robotic applications (*41*). For this reason, various studies have investigated sensor response times and worked toward improving them. Recent advancements in both hardware (*42*, *43*) and software (*20*, *44*–*50*) have substantially reduced response and recovery times from the orders of hours or minutes (*51*) down to tens of seconds or seconds (*48*, *50*). Nevertheless, those timescales remain orders of magnitudes slower than what is observed for olfactory sensing in animals, potentially stalling progress on critical challenges in tracking of greenhouse gas emissions (*52*), ecological and environmental monitoring (*53*, *54*), aerial-based wildfire detection (*55*), disaster management (*56*), and more.

In this work, we are pushing the limits of artificial olfaction with a high-speed, miniaturized electronic nose that can resolve odor pulses in the millisecond range. We propose an integrated electronic nose design of MEMS-based MOx sensors and fast sampling periphery, as well as a set of powerful algorithms for control, sensing, and signal processing. We demonstrate the systems ability to operate at unprecedented temporal timescales when classifying short odor pulses, as well as when discriminating temporal characteristics of rapidly switching odor pairs. The challenge of deploying rapid and complex odor stimuli in a controlled and precise fashion (*57*) is overcome by using a high temporal-precision olfactometer setup, which most recently has been used for showcasing the temporal odor recognition capabilities in mice (*24*, *58*).

We first elaborate on the proposed design of the electronic nose and the feedback control methods, with which we achieve thermal response times that allow for ultrafast heater cycles—orders of magnitudes faster than what is suggested in the literature. Later, we show that the electronic nose can successfully classify the odor of short pulses, with durations down to tens of milliseconds. This is achieved by rapidly switching the sensor heater temperature and then extracting phase-locked data features to train machine learning classifiers. Further, we demonstrate the system’s ability encode and infer temporal features in a task involving rapidly switching odor pairs, up to modulation frequencies of 60 Hz, which we show to match and even exceed the demonstrated capabilities of mice on equivalent tasks (*24*). This is achieved by controlling the heater temperature to be constant, permitting for sensor response feature extraction from the frequency domain. Last, we discuss our results and its implications and identify some example use cases that may benefit highly from using fast sensing modalities.

## RESULTS

### High-speed electronic nose and odor delivery system

We constructed a portable high-speed and high bandwidth electronic nose, which leverages the advantage of MEMS-based gas sensors and their rapid response times. We emphasized form factor and power consumption considerations that allow for sophisticated field measurements under space and power constraints, such as mobile robotic platforms (*34*). Our design (Fig. 1C and fig. S1A) consisted of the following elements: a microcontroller for data processing and storage, eight analog MOx MEMS gas sensors of four different types (Fig. 1D), associated analog circuitry and data converters, and a combined pressure, humidity, and temperature sensor.

Ideal MOx sensor operation requires the sensing site to be heated to several hundred degrees. The sensor response is highly dependent on the temperature and its variation over time. Previous studies have shown that a modulation of the sensor’s operating temperature often leads to better and faster gas discrimination performances (*42*), which can be traced back to physical phenomena such as transient adsorption, desorption, and diffusion processes on the sensing site (*59*). However, the suggested sensor heater cycle durations were on the orders of seconds to minutes (*42*, *49*, *60*–*63*). Aiming to achieve ultrafast heater cycles, our design couples each sensor with a separate temperature control loop, which samples the temperature and adjusts the hotplate current at high frequency. This allows to account for variations in the thermal capacities of the heterogeneous set of sensors, as well as for different airflow exposures. Further, it allows deploying different heater profiles for each sensor, which may enable targeting particular odors and specific use cases. Figure 1D shows a typical heater modulation cycle in ambient air, where the sensor resistance follows the hotplate temperature in a low-pass fashion. In our experiments, we used two different heater temperature control schemes: one that cycled between low- and high-temperature values (150° and 400°C), and one at a constant high temperature (400°C).

To provide odor stimuli to the e-nose, we used an odor delivery system that can reliably present gaseous odor samples with a bandwidth beyond 60 Hz, described earlier (*24*, *58*) and depicted in Fig. 1B. The system was based on high-speed microvalves and incorporated a flow compensation mechanism (*24*), ensuring exceptionally high temporal signal fidelity, and constant flow across the stimuli (Fig. 1, F and G, and Materials and Methods).

As prototypical, simplistic high-frequency odor stimuli, we used square pulses of different duration and separation times. A set of synthetic odorant compounds of natural food odors was considered: ethyl butyrate (EB) (pineapple), isoamyl acetate (IA) (banana), cineol (eucalyptus), and 2-heptanone (fruity/cheese). The odorants were diluted in odorless mineral oil solvent. In addition, we used two (identical) pure solvent samples as controls. The odors were presented as singular pulses with varying durations (10 ms to 1 s) and concentrations (20 to 100%) and as correlated and anticorrelated odor pulse trains (1 s) at different modulation frequencies (2 to 60 Hz).

### Rapid heater modulation enables data features robust to concentration changes

Cycling the sensor heater temperature can yield better odor classification results; however, the cycle duration may restrict the temporal bandwidth at which a stimulus can be resolved.

**Fig. 2.**
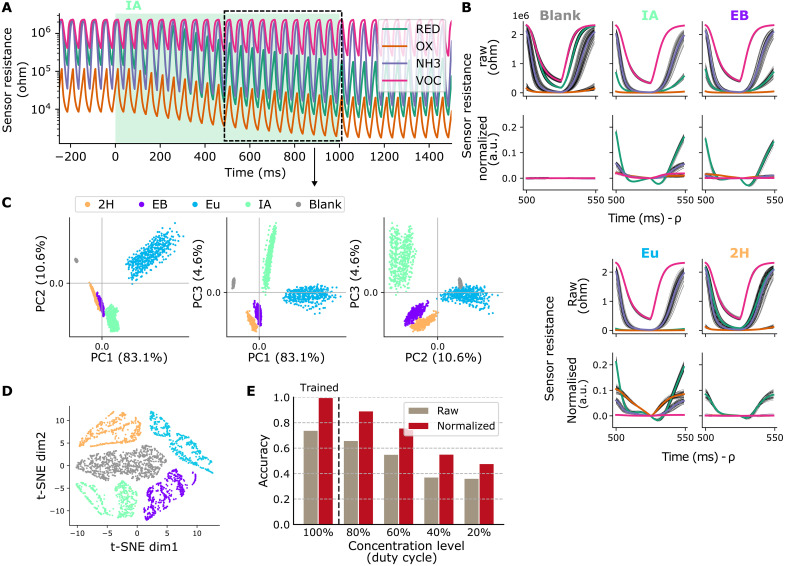
Rapid heater modulation enables robust data features. (**A**) Sensor resistance of four MOx sensors with 20-Hz hotplate temperature modulation, responding to a 1-s odor pulse of IA (green background). (**B**) Fifty-millisecond data feature for different gases, selected between 500 and 550 ms after odor pulse onset. Raw sensor response (upper) and normalized sensor response (lower; see Materials and Methods for normalization procedure). Time shifted by cycle phase ρ w.r.t. odor onset, for visual guidance only. (**C**) Principal components analysis, explained variance (most left) and projections, and (**D**) t-SNE visualization, for the set of normalized data features extracted between 500 and 1000 ms after odor onset. (**E**) Accuracy scores for a k-NN classifier trained on 50-ms data features from 1000-ms odor pulses at full concentration, and tested on 50-ms features from 1000-ms odor pulses at different concentration levels (tuned by adjusting the duty cycle of the microvalves).

In recent studies, we tested the effect of 150-ms duty cycles and found evidence for robust data features (*64*, *65*). In the current work, we leveraged our system’s ability to rapidly modulate the sensor temperature and cycled the heater temperature between a low step at 150°C and a high step at 400°C with a period of 50 ms. Notably, this is orders of magnitudes shorter than what had been suggested in previous studies (*63*). The resistance of the gas sensing elements closely tracks these changes in operating temperature (Figs. 1E and 2A), enabling us to extract gas features that are phase locked with the heater cycles for subsequent analysis and classification. For this purpose, we divided the continuous stream of gas sensor samples into 50-ms chunks aligned with the temperature cycles (Fig. 2B, upper row). The 50-ms data features further underwent prestimulus baseline normalization and scaling (Fig. 2B, lower; from now on referred to as “normalized data feature”); for details, see Materials and Methods.

For testing class discriminability and susceptibility to concentration fluctuations, we extracted data features by sampling four sensors between 500 and 1000 ms after the onset of a 1000-ms odor stimulus, for a range of concentrations. Principal component projections (Fig. 2C) and t-distributed stochastic neighbor embeddings (t-SNE) (Fig. 2, C and D) show distinct clustering that coincided with odor classes. Further, a k-nearest neighbor (k-NN) classifier was trained on data features of 1-s-long odor pulses at full concentration (100%) and tested on features of 1-s-long odor pulses at various concentrations (20 to 100%).

The classification performance results are shown in Fig. 2E. Notably, for the normalized data feature, the model provided 100% classification test accuracy at the trained concentration level, which remained at (88.7 ± 0.5)% and (81.2 ± 0.6)% when tested on 80 and 60% of the trained concentration level. Accuracy at lower concentration levels dropped substantially but remained well above chance. This is substantially better than what is achieved with the raw data feature (see Fig. 2E). Notably, while the raw data feature approaches random classification for low concentrations, the suggested normalization improves the classification accuracy substantially and thus suggests robustness to concentration changes.

### Time-resolved classification of millisecond odor pulses

In natural settings, odor bouts can be as brief as only milliseconds long. For an agent’s successful interaction with the environment, this requires the ability to classify odors fast and robustly.

**Fig. 3.**
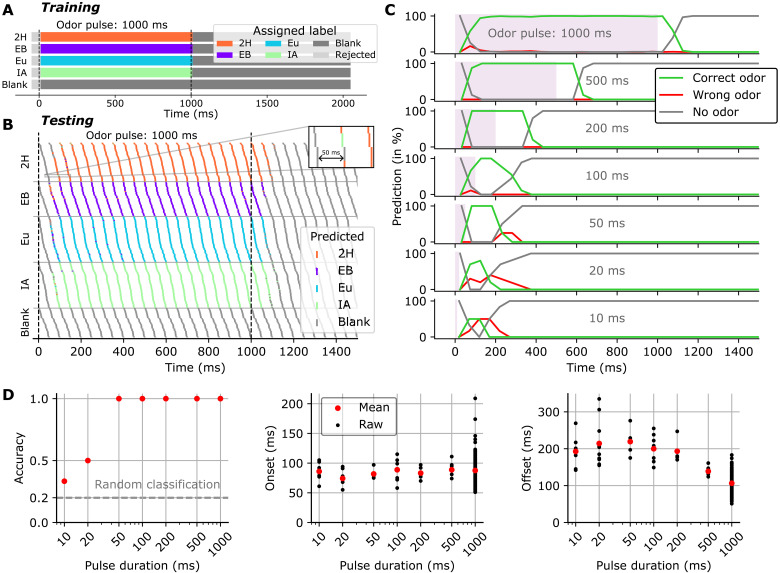
Electronic nose can classify short odor pulses on the basis of 50-ms data features. (**A**) Feature labels for the training set were phase aligned in relation to odor on- and offset. Features that overlapped with transition periods were not considered for training (“rejected”; see Materials and Methods for parameters). (**B**) Odor stimulus classification over time for odor pulses of various lengths (10 to 1000 ms), as predicted by a RBF-kernel SVM classifier trained on 50-ms features from 1000-ms second odor pulses. Shown here are 1000-ms pulses. For visual clarity only, the trials are sorted by odor, and within each odor are sorted by phase w.r.t. stimulus onset. (**C**) Classification correctness over time (evaluated via the true odor presence), for different pulse durations. (**D**) Test accuracy, onset time and offset time for the prediction over time described in (B) and (C). Onset and offset were extracted using time-to-first nonblank and blank prediction, respectively, and shown here with respect to theoretical odor onset and offset.

We evaluated the ability of the electronic nose to classify odor pulses of various durations. A support vector machine (SVM) with Gaussian radial basis function (RBF) was trained on 50-ms data features, which were acquired from eight gas sensors throughout a 1000-ms odor stimulus at five concentration levels (20 to 100%). Control trials (“blank”) were included, obtained during a 1000-ms odorless mineral oil stimulation or immediately after odor pulses. See Fig. 3A for a depiction of the labeled features. The trained model was deployed to predict the odor presence over time during exposure to odor stimulations of various durations, ranging from 10 to 1000 ms. Figure 3B displays the predicted classes over time on the example of a 1000-ms odor pulse, whereas Fig. 3C summarizes the predictions over time for all pulse durations.

From these predictions, the corresponding accuracy, onset times, and offset times were derived and shown in Fig. 3D. The classifier attained a 100% accuracy in predicting the correct class for odor pulse durations ranging from 1000 ms down to 50 ms, despite not having been trained on pulses shorter than 1000 ms. Accuracy dropped for 20- and 10-ms pulses but remained above chance level. Notably, the classifier accurately and rapidly predicted the recovery of the sensor site, indicating “no odor” when no odor was present. The time required for the classifier to correctly identify the odor remained relatively consistent across odor pulse durations, with an average value of (87 ± 20 ms). Following odor offset, the classifier robustly predicted no odor within (106 ± 24 ms) for 1000-ms odor pulses and slightly higher—yet with a higher variance—for shorter odor pulse durations.

### Decoding temporal structure of rapidly switching odors

In the presence of multiple odors, detecting whether the odor encounters are correlated or not can help to infer whether they come from the same source or from separate locations (*19*). Further, information about the encounter frequency can give rise to spatial source information (*20*). It has been shown that mice can distinguish between correlated and anticorrelated odor pairs reliably up to correlation frequencies of 40 Hz (*24*), a feat that has not yet been matched in robotic systems. Considering performance metrics based on similar tasks, here, we explored the ability of the electronic nose to resolve temporal structure of odor stimuli.

**Fig. 4.**
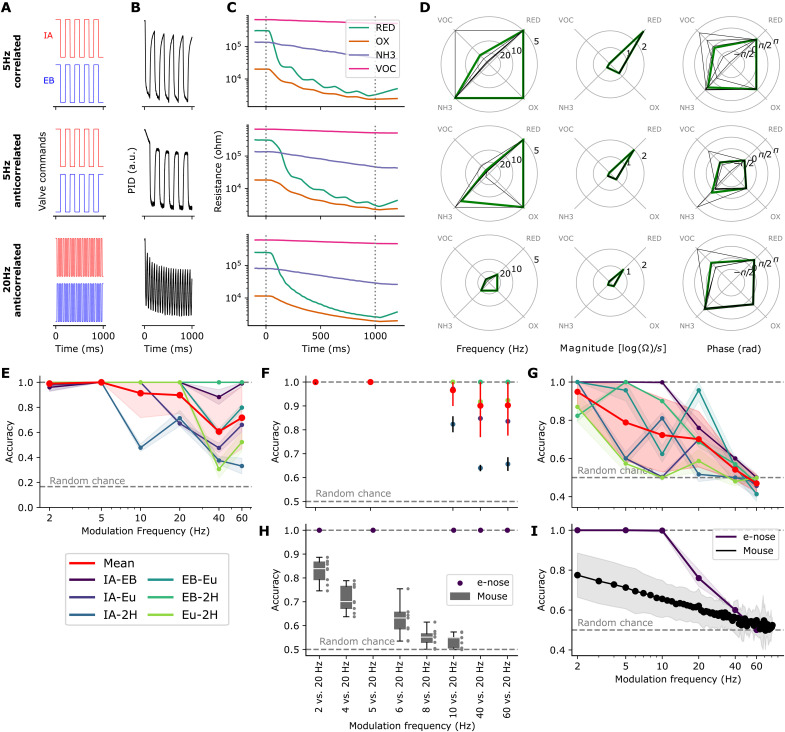
Decoding temporal structure of rapidly switching odors. (**A**) Odor valve commands. (**B**) PID response. (**C**) Electronic nose response. (**D**) Frequency, magnitude, and phase of the dominant spectral peaks. Thick lines, means of corresponding trials; thinner lines, single trials. (**E**) Accuracies for modulation frequency classification. (**F**) Accuracies for binary modulation frequency classification. (**G**) Accuracies for binary modulation mode classification (corr. versus anticorr.). (**H**) Subset of (F) for IA-EB, for mouse performance comparison [described in detail by Ackels *et al.* (*24*)]. (**I**) Subset of (G) for IA-EB, for mouse performance comparison. Panels (A) to (E) show representative trials only. For (E) to (I), electronic nose accuracy mean and SD (clipped at 1.0) arise from repeated training and testing with different random seeds.

Rapidly alternating odor pairs were presented at frequencies between 2 and 60 Hz for a duration of 1 s. We discriminated between two odor pulse trains being either in phase (correlated) or shifted by half a cycle (anticorrelated) (Fig. 4A). The resulting odor patterns follow the pulses rapidly (PID recordings in Fig. 4B).

While heater modulations lend themselves for extracting phase-locked data features and thus allowing for efficient odor classification, maintaining the sensor heater temperature constant instead allows for analyzing the data in continuous time. Particularly when observing repeating patterns or complex temporal dynamics of a stimulus, this may be advantageous, as the sensor response can be regarded in its frequency domain. Thus, for the following experiments, we operated four MOx sensors of the electronic nose under a constant hotplate temperature of 400°C. The sensors responded to the stimuli by dropping their resistance at the pulse-train onset, with the stimulus modulation visually embedded in the response (Fig. 4C). We extracted data features by differentiating and logarithmically scaling the raw sensor response, followed by a discrete Fourier transform. For each sensor, where the maximal magnitude is found, the frequency, the magnitude, and the phase were extracted. This yielded a 12-dimensional data feature (Fig. 4D). A visual comparison of the features reveals distinct differences between correlated and anticorrelated pulse trains (top versus middle), as well as between different frequencies (middle versus bottom).

On those features, ensembles of Random Forest classifiers were trained for three tasks: (i) decoding the modulation frequency of two odor pulse trains from a set of frequencies, (ii) predicting the modulation frequency of two odor pulse trains from pairs of frequencies, and (iii) decoding whether two odors pulse trains are either correlated or anticorrelated.

For the latter two tasks and a subset of the odors, a comparison with the mouse performance as detailed by Ackels *et al.* (*24*) is provided.

The test accuracies for the three tasks and all gas combinations are shown in Fig. 4 (E to G). For task 1, the data recorded with the electronic nose enable nearly perfect frequency classification (Fig. 4E) for modulation frequencies up to 5 Hz, then on average decreasing to (0.91 ± 0.20) and (0.90 ± 0.15) for 10 and 20 Hz, respectively, and lastly dropping to (0.61 ± 0.26) and (0.72 ± 0.25) for 40 and 60 Hz, respectively. For the pair-wise frequency classification (task 2; Fig. 4F), classification performance is perfect for modulation frequency pairs 2 Hz versus 20 Hz and 4 Hz versus 20 Hz, decreasing to accuracies of (0.97 ± 0.07), (0.90 ± 0.13), and (0.90 ± 0.13) for the pairs 10 Hz versus 20 Hz, 40 Hz versus 20 Hz, and 60 Hz versus 20 Hz, respectively. For discriminating correlated versus anticorrelated pulse trains (task 3; Fig. 4G), it appears that the electronic nose, on average, enables high prediction scores of (0.95 ± 0.08), (0.79 ± 0.20), (0.72 ± 0.19), and (0.70 ± 0.14) for modulation frequencies of 2, 5, 10, and 20 Hz, respectively. This drops to (0.54 ± 0.06) for 40 Hz and lastly to (0.47 ± 0.05) for 60 Hz.

In the study by Ackels *et al.* (*24*), the odor pair IA-EB has been used to test the discrimination power of fast odor dynamics in mice. In the following, we consider the corresponding subset of the electronic nose recordings and compare them to the named study. For the pair-wise frequency classification (task 2; Fig. 4H), the electronic nose classification performance is perfect for all the tested modulation frequency pairs, from 2 Hz versus 20 Hz up to 60 Hz versus 20 Hz. Here, the mouse performed substantially worse; for the pair 2 Hz versus 20 Hz, the mouse accuracy score was (0.83 ± 0.05) and then progressively dropped down to (0.53 ± 0.03) for 10 Hz versus 20 Hz. Last, considering the results from the phase prediction task (task 3; Fig. 4I), it appears that the electronic nose enables perfect prediction scores up to modulation frequencies of 10 Hz, which then steeply drops to an accuracy of (0.76 ± 0.05) for 20 Hz, (0.60 ± 0.00) for 40 Hz, and lastly to chance level for 60 Hz. In comparison, the mouse scores (0.78 ± 0.11) at a modulation frequency of 2 Hz, linearly decaying in accuracy down to chance level at around 80 Hz.

To validate whether the observed performance can be attributed to the odor signal and not to potential artifacts caused by potential hotplate temperature variations (which may be caused by unnoticed flow fluctuations), we repeated the experiment using the hotplate temperature signal (see fig. S3E), as well as the PID responses (see fig. S3I). In both cases, the analogous feature extraction and classification pipeline was performed, resulting in classification performances as displayed in fig. S3 (F to H) for the hotplate temperature and fig. S3 (J to L) for the PID responses. The analysis confirmed that there was not enough information in the hotplate temperature response alone to classify the odorants with above chance performance. Further, using the PID response, which should be unaffected by potential flow fluctuations and is commonly used as a ground-truth measurement, nearly perfect accuracy scores were achieved for most gas combinations across the tested tasks.

## DISCUSSION

For many tasks and applications in robotics, natural and turbulent environments pose the challenge of highly dynamic and rapidly changing odor concentrations, which demands high temporal resolution odor sampling and processing. Intrigued by the exceptional speed at which animals process and respond to odors, we challenged the limits of artificial olfaction by demonstrating a portable electronic nose that achieves the temporal resolution of mouse olfaction. The high temporal resolution we achieved enables a new perspective on resolving temporal dynamic olfactory stimuli in turbulent environments, such as those encountered by agents (animals and robots) in natural environments. Achieving this required the following: (i) Compact high-performance electronic nose: We designed a high-bandwidth, high-resolution electronic nose using modern MEMS gas sensors with minimal thermal mass, which allows for rapid heat transfer to and from the analyte odor. We selected sensors with an analog interface, included high-end analog-to-digital converters (ADCs)/digital-to-analog converters (DACs) that robustly sampled the sensors at 1 kHz, and engineered the printed circuit board (PCB) layout to minimize noise and cross-talk. (ii) Advanced heater control and data features: We implemented a combination of open-loop and adaptive closed-loop control algorithms for precise heater control. In addition, we designed and optimized novel data features for specific tasks. For short odor pulse classification, we created heater phase-locked data features and normalized them to reduce dependency on the baseline response. For decoding temporal signal characteristics, we used log normalization and fast Fourier transform (FFT) to access frequency space and select dominant frequency features. These features were used to train and evaluate machine learning classifiers on the respective task. (iii) Rigorous temporal evaluation: We conducted an extensive evaluation of the electronic nose’s temporal capabilities using a sophisticated experimental setup and protocols akin to those in a recent landmark olfaction study, involving ultrafast odor valves and short gas paths for high-fidelity odor pulse delivery. 

Through these experiments, we demonstrated that the electronic nose can successfully infer the odor identity of single-odor pulses down to durations of 10 ms, albeit being trained on 1-s odor pulses only. This was achieved through modulating the sensor temperature with cycle periods of 50 ms, extracting and preprocessing the corresponding sensor response and then training and evaluating a classifier.

Further, we demonstrate the electronic nose’s ability to predict whether two-odor pulse trains were correlated or anticorrelated up to switching frequencies of 40 Hz, matching or exceeding mice on the equivalent task. For tasks involving determining the odor switching frequency (multiclass and binary), we demonstrate a high performance up to 60 Hz, outperforming mice on equivalent tasks. For this, the sensor heaters were set to provide a constant temperature, which allowed an analysis of the data in the frequency domain.

An interesting discussion arises on why fast heater modulations lend themselves so well for fast odor pulse classification. We see two main reasons: (i) Heater modulations generally result in much faster sensor responses compared to isothermal operation (*42*). This is because the sensors’ conductive behavior, influenced by adsorption, desorption, diffusion, and reaction processes, varies substantially with temperature (*59*). By cycling the sensor through a range of temperatures, transient processes within these phenomena are driven, producing responses that are highly characteristic of a given gas. This creates a more nuanced mapping of the sensor response, capturing a wide range of temperature-dependent behaviors specific to each odor in a short time. (ii) From a signal processing perspective, coupling sample windows to the phase of heater modulations creates natural, multidimensional units of computation. Each data window begins at the same temperature and follows the same temperature range, akin to active sensing. This approach results in consistent data features, which improves machine learning classifier performance by allowing for a well-separable feature space and thus for robust odor classification.

Further insights on task-specific sensing modes and sampling can be gained from those results. First, data windows that are phase locked with ultrashort sensor temperature cycles appear to be a sensible choice when given the task of odor classification when the stimulus is short. The high sampling rate allows for a multidimensional data feature (here, four or eight sensors times 50 samples per feature), which, together with the prestimulus normalization procedure, successfully captures the odor-specific sensor response. In mammalian sensory neuroscience, the analogy would be the phase coupling of spike trains to the inhalation cycle, allowing the spike timings to encode information about the odor identity and thus suggesting one “sniff” as the unit of olfactory processing (*66*). Conversely, if the task is not classification but decoding temporal information about the odor stimuli, such as frequency or correlations, integrating the information across an artificial time window would limit the temporal resolution; hence, recording continuously and without heat modulation might be the better choice. The ability of mammals to access temporal stimulus information at subsniff resolution has been demonstrated (*24*, *58*) and shown to be relevant for behavioral tasks (*24*). Those findings may suggest future experiments in which both modes are active simultaneously on separate sensor instances—continuously sampled constant-temperature and time-integrated temperature-modulated—which could allow for extracting information about the temporal profile and the identity in parallel. Research on insect have suggested dual-pathway olfactory systems (*67*), which may facilitate the simultaneous extraction of odor identity and concentration information (*68*). Such an approach might suggest elegant solutions to the olfactory cocktail party problem (*69*–*71*).

The proposed experimental approach appears to be well suited to verify and characterize the temporal capabilities and in particular the high-frequency properties of the device. Yet, it presents limitations when considering its evaluation with respect to more natural environments. The current setup, characterized by fast valves, short odor delivery lines, and precise flow compensation, does not necessarily replicate the turbulent and intermittent nature of odor plumes encountered in typical environments, where odor packets are less sharp and potentially of lower concentration. This should motivate further experiments that take into account scenarios that resemble natural environments more realistically. When designing such, it will be crucial to ensure that the setup allows for both accurately replicating the turbulent and variable nature of odor plumes and carefully controlling or monitoring the ground-truth odor concentrations. In such, it will be important to use methods that do not interfere with the natural flow characteristics, which, e.g., for PID concentration monitoring is a known challenge (*72*), and which, e.g., Planar-laser-induced-fluorescence monitoring (*73*) would allow for under certain constraints. Ensuring that these conditions are met will help validating the robustness and adaptability of the electronic nose in real-world environments, thus providing a more comprehensive assessment of its performance. Further, for the odor classification data features to be fully applicable to dense odor stimuli, it may require altering the prebaseline normalization, i.e., replacing the fixed prestimulus distance with, e.g., a moving average.

The proposed technology and its evaluation hold promise for tackling many real-world challenges that require rapid odor sensing. In particular, any instance of olfactory robotic solutions might currently be cut short in terms of performance; as for both UGVs and UAVs, the sensor response time dictates the maximum speed at which the agent can move while still obtaining spatially resolved measurements (*53*). Thus, such applications may directly benefit from using the proposed sensor modalities, allowing for faster identification and localization of odor sources.

For instance, a recent work proposed swarms of nano quadcopters performing gas source localization in indoor environments (*74*) and evaluated different search strategies. A decreased latency in detection and classification may assist not only in more efficient source localization but also in expanding the use case to multiple odors and more complex outdoor environments. Another recent study proposes odor sensing on drones for wildfire monitoring (*55*). For detecting smoke, vision and gas sensors are fused; however, the gas sensor update frequency is just 1 Hz. Given the intermittent and fine-structured nature of odor plumes, an improvement on sensing timescales could reduce false negatives and aid in gaining critical time in localizing the fire. Further studies have proposed interfacing living insect antenna tissue with robotics to alleviate the slow sensor response of traditional odor sensor systems (*43*, *75*–*77*). Using the electroantennogram has proven effective, allowing for response times of up to 10 Hz (*43*). However, this “bio-hybrid” approach carries the burden of maintaining the tissue samples alive, which is currently not feasible beyond a few hours (*76*). Here, we would propose an electronic nose as a more sustainable solution, as the MOx sensors used in this system have been industrially tested for continuous use over several months to years.

Beyond robotics, most applications in security still use static and relatively slow sensing platforms, e.g., the odor-based detection of explosives (*78*) at airports. At checkpoints, fast and portable electronic noses could replace random spot checks with exhaustive controls and thus minimize risk further. Further, recent investigations on mammalian olfactory-guided behavior use head-mounted MOx sensors as control recordings (*79*). Using a high-resolution data acquisition system—particularly one that matches the temporal capabilities of the subject—would allow for better data quality and hence could improve the resulting models.

In a related vein, neuromorphic information processing (*80*, *81*) has seen much traction in recent years, where in particular the reduced latency, power consumption, and data bandwidth have enabled highly optimized vision and auditory sensors (*82*, *83*). We suggest that the information embedded in millisecond odor packets, together with the sparse and intermittent nature of odor plume encounters, makes the sense of olfaction an ideal candidate for neuromorphic sensing. We foresee that revealing the rapid nature of the sensors will further stimulate this field of research, motivating event-driven and asynchronous odor sampling (*84*), for MOx sensors (*85*, *86*) and beyond (*87*, *88*).

In conclusion, our study marks a groundbreaking advancement in electronic olfaction systems, demonstrating the ability to discern odors and decode odor patterns with unprecedented temporal precision in miniaturized low-power settings. Our findings unlock new possibilities for developing robotic systems capable of rapidly and precisely tracking odor plumes in compact and low-power environments, with the potential to transform electronic nose designs and their applications across various domains.

## MATERIALS AND METHODS

### Electronic nose

#### Circuit board design

The electronic nose uses readily available components and is illustrated in fig. S1A. It features a Raspberry Pi Pico microcontroller and incorporates eight MEMS-fabricated MOx gas sensors of four different types, grouped into four packages.

The sensor packages comprise two ScioSense CCS801 sensors (sensors 1 and 5) and two SGX Sensortech MiCs-6814 sensors (sensors 2, 3, 4 and 6, 7, 8), capable of detecting a wide range of reducing and oxidizing gases, including volatile odor compounds, hydrocarbons, carbon monoxide, hydrogen, nitrogen oxides, and ammonia. The sensors were manufactured using MEMS processes and are small, fast, and ultralow power. The integrated microhotplates allow for operation at temperatures of up to 500°C.Their small thermal capacity allows for rapid heating and (passive) cooling. Figure 1C displays an optical microscopy image of the sensor structure. Previous studies suggested decapping the gas sensors for increased airflow and reduced latency (*43*). For our experiments, we did not decap the sensors, as we wanted to minimize the risk of sensor damage and contamination, as well as to ensure reproducibility.

For controlling the microhotplates, eight low-noise and low-distortion operational amplifiers (2x STMicroelectronics TS924) are used. To configure the heater voltages, we use two DACs, specifically the internally buffered and ensured monotonic Texas Instrument DAC60004, offering four channels each at 12 bits and 1 kHz, and a high linearity of less than one least significant bit.

In addition, two ADCs, the Texas Instrument ADS131M08, are used to read sensor and heater resistances. These are differential, simultaneous-sampling ADCs, which read out the eight gas channels and the eight temperature channels in lockstep at 24 bits and 1 kHz (the two ADCs share the same clock). To monitor environmental conditions, a digital pressure-humidity-temperature sensor, the TE Connectivity MS8607, is included, which samples data at 24/16/24 bits and 50 Hz. Real-time data logging is facilitated through the inclusion of a microSD card. The four-layer PCB design has been designed following best practices in mixed analog design to minimize noise and cross-talk. The device’s power needs, ranging from 1.2 to 1.5 W, allow for multiple days of continuous operation on a pocket-sized battery pack, making it suitable for extended field recordings or robotic environments.

#### Sensor heater modulation

##### Heater temperature read-out

To implement controlled heater modulation, continuous measurement of the hotplate temperature and regulation of power delivered to the resistive heating element are essential. Each heater voltage *V*_heat_ was adjusted using a DAC and an associated amplifier, while the resulting current *I*_heat_ was monitored using an ADC in conjunction with a fixed-value sense resistor *R*_sense_. From these two quantities, one can compute the heater resistance *R*_heat_ = *V*_heat_/*I*_heat_ and dissipated power *P*_heat_ = *V*_heat_*I*_heat_. Because the device did not directly measure *V*_heat_, the resistance calculated by substituting the known control and sense voltages *V*_DAC_ − *V*_sense_ ≈ *V*_heat_ was subject to errors, of which transient errors caused by lag and settling time in the DAC and amplifier were deemed the most significant. These affected the sample acquired immediately after a change in control voltage; therefore we used a Kalman filter (*89*) to estimate *R*_heat_, setting the measurement uncertainty proportionally to the rate of change of the control voltage *V*_DAC_.

##### Open-loop and closed-loop control

Our design couples each sensor with a separate temperature control loop. This allows for addressing the different thermal capacities of the heterogeneous set of sensors and thus counteracts under- and overshooting of each heater temperature, a phenomenon that may be expected if a single control loop were used to modulate the average temperature across all heaters. Further, it allows for sophisticated heater protocols—such as having different heater cycles for different sensors or having some sensors run at constant heat and others modulated—which may allow targeting particular analyte gases and/or specific use cases.

Achieving short temperature steps of a duration not much greater than the thermal time constant of the hotplate presented a number of challenges. Because the shortest steps consisted of only 25 samples (25 ms at 1 kHz), we opted against the commonly used proportional-integral-derivative controller, as such does not guarantee optimal control or stability, and is known to be particularly susceptible to system lag and nonlinearities (*90*). Instead, we used a combination of open-loop (feed-forward) and closed-loop control, on which we elaborate in the following:

The kind of resistive heating element present in our design exhibits a quasi-linear relationship between hotplate resistance and temperature (*91*). Thus, as a first approximation, we used a linear open-loop model that maps the heater resistance to a calculated hotplate temperature. This model is created and parameterized during a calibration protocol, in which the temperature response to a set of power steps is measured, and matched with calibration data from the manufacturer datasheet, namely, the nominal hotplate temperature delta above ambient air temperature at nominal heating power. The calibration protocol and model parameterization has been run shortly before a set of experiments and should partially take into account the state (i.e., age, contamination/poisoning, and long-term drift) of the sensor as well as environmental conditions.

In addition to the open-loop control, a closed-loop control function is implemented to (i) minimize any mismatch between the target temperature and the actual temperature caused by, e.g., nonlinearities that the linear feed-forward part cannot address and to (ii) compensate for perturbing effects caused by fluctuations in airflow, ambient temperature, humidity, etc. In the closed-loop part, we measured the hotplate temperature at every step, computed the error between the measured temperature and the target temperature, and added the error to the control signal via a fixed adaptation rate (or learning rate), which in most cases was fixed at 0.1 V °C^−1^ s^−1^. This resembles a simple proportional controller, which in this case is enough, because the open-loop control already brings the temperature in the right regime.

In a small ablation study, we investigated the efficacy of this control scheme in modulating the heater temperature. At ambient temperature and with zero air flow, we ran the initial calibration protocol of the electronic nose, which would create the mapping that is later used for the feed-forward control. Then, we ran the sensors either at constant 400°C or at 25-ms temperature cycles between 200° and 400°C allowed for a warm-up period of 2 min and started recording the sensor hotplate temperatures for four sensors at *t* = 0 s. At *t* = 120 s, we activated the airflow, which in this case was affected by turbulence, and not as focused as in the other experiments described in the manuscript. At *t* = 240 s, we deactivated the airflow and recorded until *t* = 360 s. We repeated this experiment once with just open-loop control and once with the proposed scheme open-loop + closed-loop. The resulting measurement traces are shown in fig. S1G. It is evident that the combination of open-loop and closed-loop is advantageous compared to open-loop alone, both in bringing the measured temperature close to its set point and in stabilizing the temperature during and after air flow perturbances. It is important to note that the here shown change in airflow is an extreme case and not something we encountered in the main experiments described in the paper. There, the airflow was compensated by the odor delivery system, resulting in virtually zero flow perturbations.

##### Constant heat

For experiments with a constant heater temperature, achieving fast temperature changes was not an issue, but care was taken to avoid the artifacts caused by the DAC’s 12-bit quantization of applied heater voltage. These quantization steps of about 0.7 m led to small but measurable transients in the recorded sensor signal. Because these transients were in the same frequency band as the signals of interest, we decided to also keep the heater voltage constant during each stimulus. We adjusted it to eliminate the temperature error after each stimulus, which was sufficient because the thermal environment of the sensors changed only slowly.

#### Sensor responses to ambient air

The resistance of MOx sensors depends not only on the presence of gases but also on the operating temperature. In heater cycle mode and odorless air (see Fig. 2B, left), the sensors exhibited nearly exponential relationships between the recorded sensor resistances and the hotplate temperatures in the range of 200° to 400°C, with deviations at lower temperatures (see fig. S1C). A small deviation can be observed between the trajectories corresponding to heating and cooling; however, this may be attributed to uncertainties in estimating the temperature of the sensor, which is close to but not necessarily equal to that of the hotplate. The resistances returned to their initial values after a completed cycle, without significant hysteresis.

### Odor delivery setup

#### Odorant selection and reagents

In our study, we wanted to provide a fair comparison of the electronic nose with the mammalian olfactory system. Thus, we selected a set of odorants that was used in the recent study investigating the temporal capabilities in mammals (*24*). Here, we used dilution factors that were equal or higher than the reported ones (*24*). All used odorants are synthetic compounds of natural food odors, with smells that are perceived as pineapple-like (EB), banana-like [IA, eucalyptus-like (eucalyptol/cineole), and fruity or cheese-like (2-heptanone). While chemically similar—they are all organic compounds made up of carbon, hydrogen, and oxygen atoms—the set contains two esters, one ether and one ketone. Relevant chemical properties are summarized in Table 1. All odorants were obtained in their pure liquid form from Sigma-Aldrich and contained in 15-ml glass vials (27160-U, Sigma-Aldrich). They were diluted using an odorless mineral oil that, in its gas phase, would not react with the sensors and is not detectable by mammals.

**Table 1.** Chemical properties of selected odorants. Data from https://pubchem.ncbi.nlm.nih.gov/.

Name	Formula	Class	Molecular weight (g/mol)	Vapor pressure (mmHg at 25°C)	Dilution (%)
Isoamyl acetate	C_7_H_14_O_2_	Ester	130.18	5.6	20
Ethyl butyrate	C_6_H_12_O_2_	Ester	116.16	11.3	20
Eucalyptol / cineole	C_10_H_18_O	Ether	154.25	1.9	20
2-Heptanone	C_7_H_14_O	Ketone	114.19	3.85	5

#### Olfactometer

Odors were presented using a custom made olfactometer capable of constructing temporally complex stimuli with temporal bandwidths of up to 500 Hz (Fig. 1D). This temporal olfactory delivery device (TODD) has been outlined previously (*24*, *58*). The device consisted of eight independent channels that contained either odor (diluted with mineral oil) or pure mineral oil. These eight channels were grouped into two sets of four. Each set of four consisted of an odor manifold, which contained odors or pure mineral oil in glass vials that were fed by a common air flow. Each channel in this odor manifold was fed into its own high speed valve on a separate valve manifold. Each high speed valve could be opened and closed at frequencies of up to 500 Hz. On each valve manifold, one of the channels containing pure mineral oil was set to remain open indefinitely, acting as a “carrier” valve (gray valves in Fig. 1D). When a trial was triggered, this carrier valve flow was reduced in accordance with the amount of additional airflow generated by the other valves on the manifold, therefore maintaining a continuous rate of air flow through the system. In some cases, the carrier valve was not simply reduced but was used to generate temporally complex airflow to compensate for the temporal patterns generated using the other valves in the system. Signals to the valves were convolved with a high-frequency 500-Hz continuous signal, referred to as “shattering.” This shattering was included as it has been previously found to improve the temporal fidelity of the resultant odor signal. The airflow to the TODD was maintained at a rate of 1 liter/min using a custom closed-loop-feedback flow controller.

#### Calibration

To ensure a continuous total airflow whilst maintaining a high signal fidelity, before the electronic nose recording session, the output of the olfactometer was measured with both a PID (200B miniPID, Aurora Scientific) and flowmeter (AWM5101VN, Honeywell). The PID was positioned a short distance away from the output of the olfactometer (2 cm), and calibration trials were presented. These were selected in a way to making sure to cover all the different valve combinations of the experiment. The PID response to presented odors was measured and the fidelity estimated. If the fidelity was found to be too low, the rate of flow into each channel was tuned to increase the fidelity. Next, the PID was replaced with the flowmeter, and the same selected calibration trials were presented. If the rate of flow varied during the trial presentation, the compensatory flow or carrier flow was modulated to return the net flow back to pretrial levels. The flow through the odor valve was kept constant so as to not alter the odor signal fidelity. Airflow was modulated by altering the duty-cycle of the valve shattering. Once there was no visual change in the rate of flow between the trial and pretrial levels, the flowmeter was removed and the olfactometer was deemed to be calibrated.

#### Fidelity calculations

For quantifying the olfactometers’ temporal fidelity after calibration, we deployed single-odor pulse trains of different frequencies and obtained simultaneous PID and flow meter recordings. Here, the odorant EB was used, as its ionization energy is well suited for the used PID. In particular, at *t* = 0 s, the carrier flow valves opened while odor valves remained closed, for a duration of 10 s. At *t* = 10 s, the odor valve and odorless compensation valve deployed anticorrelated pulse trains of various frequencies, for 2 s [see fig. S1A (left) for the 10-Hz example]. The fidelity for each square pulse was calculated as the value of peak to trough, normalized to the peak to the baseline value. The fidelities shown in fig. S1A (right) were computed as the mean and standard-deviation across all square pulses fidelities of a particular modulation frequency.

### Experimental protocol

#### Electronic nose placement

The electronic nose was attached to a movable arm and fixed in place downstream of the olfactometer outlet, with a distance of ~3 cm from outlet nozzle to the gas sensors. To ensure that the gas flow reached all the sensors on the board, we fine-tuned the alignment of the electronic nose with respect to the nozzle by trial and error until a strong response was obtained on all channels.

#### Heater modulation and odor delivery protocol

Three experiments (A to C) with different sensor heater conditions were performed. See Table 2 for the details and fig. S1D for an illustration. For each experiment, odor stimuli of different pulse widths and concentrations (controlled by adjusting the shattering duty cycle of odor and mineral oil valves) were presented. After each odor stimulus, there was a 30-s recovery phase before the next stimulus onset. The odor delivery protocols used for the analysis in this work are listed in Table 3.

**Table 2.** Sensor temperature conditions.

Experiment	Sensor	Condition	Temperature range (°C)	Comment
A	1–8	50-ms cycles	150–400	-
B	1–4	Constant	400	-
	5–8	50-ms cycles	150–400	-
C	1–4	Constant	400	-
	5–8	200-ms cycles	150–400	Not used

**Table 3.** Odor delivery protocols Acorr. PT., anticorrelated pulse trains; Corr. PT., correlated pulse trains.

Kind	Odors	Duration (ms)	Frequency (Hz)	Concentration (%)	Trials
Pulse	4 + 2	1000	-	100	300
Pulse	4	1000	-	80	80
Pulse	4	1000	-	60	80
Pulse	4	1000	-	40	80
Pulse	4	1000	-	20	80
Pulse	4	500	-	100	20
Pulse	4	200	-	100	20
Pulse	4	100	-	100	20
Pulse	4	50	-	100	20
Pulse	4	20	-	100	20
Pulse	4	10	-	100	20
Acorr. PT.	4	1000	2	100	60
Acorr. PT.	4	1000	5	100	60
Acorr. PT.	4	1000	10	100	60
Acorr. PT.	4	1000	20	100	60
Acorr. PT.	4	1000	40	100	60
Acorr. PT.	4	1000	60	100	60
Corr. PT.	4	1000	2	100	30
Corr. PT.	4	1000	5	100	30
Corr. PT.	4	1000	10	100	30
Corr. PT.	4	1000	20	100	30
Corr. PT.	4	1000	40	100	30
Corr. PT.	4	1000	60	100	30
				**Total**	**1280**

Within each experimental run, all the stimuli were presented in a fully randomized order. Figure S1E shows the distribution of odors over time, binned in 1-hour time intervals. A statistical chi-square test was performed, confirming that the null hypothesis cannot be rejected (*P* = 0.364), i.e., that the trials are in fact randomized. The odor delivery protocol has not been synchronized with the sensor heater modulation phase.

#### PID recordings and odor onset/offset determination

A shortened version of the odor delivery protocol was deployed and recorded with the PID. Figure S1E displays a PID response to a 1-s IA pulse. For all the odors, the mean and SD of the prestimulus baseline were computed, and a threshold of four times the SD (4σ) defined. This was used to estimate an upper bound for the time from theoretical stimulus onset to odor exposure at the sensing site and a lower bound from theoretical stimulus offset to the purging of the sensor site. Figure S1F displays all the extracted onset and offset values, indicating that the odor may reach the sensor as rapidly as in 10 ms, while the purging may take several hundreds of milliseconds. While PIDs are extremely fast, they too have a finite and odor-dependent response time; thus, the actual times may be shorter than this.

### Pulse classification analysis

#### Feature extraction

For evaluating which data features may be most suitable for the rapid classification of short odor pulses and for evaluating its robustness to concentration fluctuations, we used experiment B, where sensors 1 to 4 were operated at a constant temperature of 400°C, and sensors 5 to 8 used heater cycles of 50 ms in the range of 150° to 400°C.

Data windows of 50 ms starting at a given time *t* relative to the stimulus onset at *t*_onset_ = 0 s were used to extract (i) raw data from constant heater sensors, (ii) prestimulus (*t*_pre_ = −5 s) baseline subtracted data from constant heater sensors, (iii) raw data from cycled heater sensors, and (iv) prestimulus (*t*_pre_ = −5 s) baseline normalized data from cycled heater sensors. For the normalization in the latter, the procedure is illustrated in fig. S2D and described in the following. The extraction of the feature $G\left(D_{s},t\right)$ can be summarized as applying a chain of a sensor-wise logarithmic transformation and a maximum scaling, to both a 50-ms baseline data snippet before stimulus onset (here, *t*_pre_ = −5 s) and to a snippet at time *t*, and then computing their vector difference

$$G\left(D_{s},t\right)=\frac{\text{log}\left[H_{\text{cycle}}\left(D_{s},t\right)\right]}{\text{max}\left\{\text{log}\left[H_{\text{cycle}}\left(D_{s},t\right)\right]\right\}}-\frac{\text{log}\left[H_{\text{cycle}}\left(D_{s},t_{\text{pre}}\right)\right]}{\text{max}\left\{\text{log}\left[H_{\text{cycle}}\left(D_{s},t_{\text{pre}}\right)\right]\right\}}$$ (1)

Here, $H_{\text{cycle}}\left(D_{s},t\right)$ describes a kernel extracting data from the sensor recordings $D_{s}$, using a window that begins at time *t* and ends after one full heater cycle (e.g., 50 measurements). The sensor index is denoted as *s*. The prestimulus normalization is a necessary and commonly used step that eliminates the first-order sensor baseline (i.e., here the data 5 s before the stimulus), which is known to often be contaminated the effects of sensor drift and fluctuations in environmental conditions (*92*).

#### Static pulse classification

##### Data splitting and model selection

The data were split into one set for training and validation and one set for testing—with a ratio of 60 to 40%. See fig. S2A for a schematical overview of the data splitting heuristic.

The former data split was used to train and validate a k-NN classifier using different features via cross-validation. The latter was used to evaluate/test the performance with the different features. We selected the k-NN algorithm for this task in particular, as it is one of the simplest nonparametric method that offers robustness to noise in the data.

##### Model training

Classifier training was performed on data features from sensor responses between 500 and 1000 ms after stimulus onset, where the stimuli were 1000-ms odor pulses of the gases 2H, EB, IA, Eu, and blank, at 100% concentration.

##### Model validation and optimization

For this task, we used fivefold cross validation on the training and validation split, which splits the data further into contiguous batches, from which four are used to train the model and one to obtain validation scores. Because the classes were balanced, i.e., all contained an approximately equivalent number of samples, it was sufficient to consider the achieved accuracy scores. Because k-NN is not parameterized, it was not necessary to optimize the model beyond selecting the number of neighbors, which in this case we selected as the number of different classes, i.e., 5.

Further, on the validation batches, we also included stimulus concentration sampled in the range [20, 40, 60, 80]%, while omitting the blank class. We repeated the evaluation using accuracy scores and confirmed that the model performs well in these cases.

##### Model testing

Testing was performed on equivalent data features; however, now for all the stimulus concentrations together, i.e., [20, 40, 60, 80, 100]%, while again omitting the blank class. Figure S2D displays the achieved performance using the different data features. The normalized cycled-heater data feature $G\left(D_{s},t\right)$ outperforms the other tested features, both in accuracy at 100% concentration, as well as for the reduced concentrations. For clarity, Fig. 2E shows a subset of fig. S2D.

#### Dynamic pulse classification

##### Data splitting and model selection

For the dynamic classification of short odor pulses, data features were again extracted as described in Eq. 1. Experiment A was used with all eight sensors modulated on a 50-ms period between 150° and 400°C. Again, a 60% versus 40% split for training and validation versus testing was performed, where the former was used to determine a suitable classifier and its hyperparameters via cross-validation, while the latter served to evaluate the performance of the classifier (see fig. S2B).

##### Model training

For training, the underlying data for the features are the sensor responses for the subsequent 2000 ms after the onset of 1000-ms odor stimuli, for concentrations in the range [20, 40, 60, 80, 100]%. The data features were labeled according to their time *t*, as described in Algorithm 1.


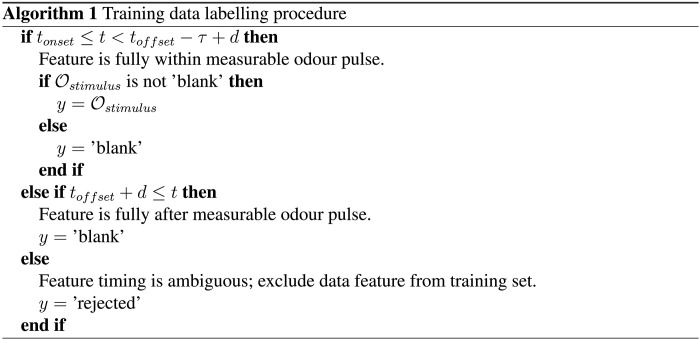


Here, *t*_onset_ = 0 ms and *t*_offset_ = 1000 ms are the stimulus onset and offset, respectively, τ = 50 ms is the feature duration, *d* = 10 ms is the upper bound stimulus delay as computed earlier, $O_{\text{stimulus}}$ is the stimulus odor of the corresponding trial, and *y* is the prescribed label of the data feature in question. This procedure is illustrated in Fig. 3A.

##### Model validation and optimization

For the normalized data features, several classification algorithms were trained and validated via fivefold stratified cross-validation, analogously to the previous task. The best performing algorithm with corresponding hyperparameters was selected, which here was a SVM classifier with RBF kernel (*93*) (*C* = 1*e*3, γ = 1 × 10^−4^, balanced class weight). Ultimately, an ensemble classifier was composed from the five SVMs trained on each split. RBF-SVM algorithm is a reasonable choice for this task in particular, as it is guaranteed to find the hyperplane that maximizes the margin between classes. Thus, we can expected better generalization on unseen data than simpler (e.g., kNN) algorithms. The RBF kernel allows for considering nonlinearities in the data, despite the natively linear nature of the SVM algorithm.

In addition, on the validation batches, we included shorter odor pulses with stimulus durations within the set {10, 20, 50, 100, 200, 500, 1000}ms. We repeated the evaluation using accuracy scores, and confirmed that the model performs well in these cases.

##### Model testing

For testing how well the trained classifier performs on shorter odor pulses, the features were extracted from sensor response data for the subsequent 2000 ms after the onset of odor stimuli of different durations, at 100% concentration. The stimulus durations fall within the set {10, 20, 50, 100, 200, 500, 1000}ms. For each data feature, the classifier predicted the odor, which is illustrated as a raster plot in Fig. 3B. The predicted odors *y* were compared against the actual stimulus odor $O_{\text{stimulus}}$ and divided in predicting “correct odor,” “wrong odor” and “no odor,” resulting in Fig. 3C. To extract the accuracy for each pulse duration, a confusion matrix was composed by—for each trial—comparing the most predicted nonblank class against $O_{\text{stimulus}}$, across multiple trials. The on- and offset times correspond to the elapsed time from odor onset to first nonblank prediction and from odor offset to first blank prediction, respectively. An analogous procedure was followed for testing the trained classifier on anticorrelated patterns of odor pairs, resulting in predictions over time, as shown in fig. S2E.

### Temporal structure analysis

#### Feature extraction

For the temporal structure analysis, i.e., the determination of the frequency and the phase-shift of the two-odor pulse trains, the constant heater sensor data (i.e., sensors 1 to 4) of experiments B and C were used. In particular, experiment B was used for training and validation (i.e., finding and evaluating a suitable data feature and classification algorithm), where experiment C was used for testing, see fig. S3A for an illustration.

For each data trial, we extracted sensor data $D_{s}$ from *t* = *t*_onset_ to *t* = *t*_offset_ + *b*, where *b* = 100 ms to account for the stimulus delay and potential sensor lag. The data was then log transformed and differentiated before applying a discrete Fourier transformation F(.), using the FFT algorithm (*94*)

$$L\left(D_{s}\right)=F\left[\frac{d}{dt}\text{log}\left(D_{s}\right)\right]$$ (2)

All triplets [frequency, magnitude, phase] were extracted, and sorted according to the magnitude. For each of the four sensors, the triplet with the highest magnitude was selected, collectively composing a 12-dimensional data feature.

#### Frequency and correlation decoding tasks

##### Data splitting and model selection

As for the previous tasks, the data were split into one set for training and validation, and one for testing. Figure S3A illustrates the following splitting scheme: Experiment B was used for training and validation (i.e., finding and evaluating a suitable data feature and classification algorithm), whereas experiment C was used for testing.

We decided on using a Random Decision Forest (RDF) classifier (*95*) (balanced class weight, *N*_tree_ = 100), and on using the same 12-dimensional data feature for all tasks. RDF was used here, as it allows considering interdependencies of the data features dimensions, which is suggested by the inherent coupling of the FFT peaks’ frequency, magnitude, and phase.

##### Model training, validation, and optimization

The data features and potential classifiers were evaluated on a 10-fold cross-validation using the training and validation data (i.e., experiment B) and the three different tasks described earlier. In particular, for each cross-validation training batch, we trained a RDF classifier on different data features, and evaluated it on the validation batch by considering accuracy scores. We did not further optimize the RDF model and used the default number of estimators as *n* = 100. Last, we combined the RDF models to form an ensemble classifier for each task. See fig. S3 (B to D) for the validation results of the data features.

##### Model testing

The RDF ensemble was ultimately evaluated on the testing data (i.e., experiment C). For the evaluation using hotplate temperature and PID data, the analogous pipeline was used, except that we omitted the log transformation.

### Comparing electronic nose performance with mouse performance

Performance analysis of the electronic nose to discriminate odor correlation structure was carried out in the style of a previously published experimental dataset [see the study by Ackels *et al.* (*24*)]. This allowed for a direct comparison of the electronic nose performance with that of mice during an operant conditioning task. A complete description of the experimental conditions and data analysis can be found in the original paper. In brief, two cohorts of up to 25 mice were housed in a common home cage system (*96*) that is used as an automated operant conditioning setup. Mice were trained to discriminate perfectly correlated from perfectly anticorrelated odor stimuli switching at frequencies ranging from 2 to 81 Hz. Task frequency was randomized from trial to trial. Odors were presented with a multichannel high-speed odor delivery device similar to the one used in this manuscript. During a go/no-go task animal performance was rated on the basis of their lick responses to *S*+ (rewarded) and *S−* (unrewarded) stimuli. For roughly half of all mice, the correlated pattern was *S*+ and the anticorrelated pattern was *S−*. In the other half of the group, this reward valence was reversed. All stimuli were 2 s long. A water reward could be gained by licking so that licking was detected for at least 10% of the stimulus time during an *S*+ presentation (a “Hit”). Licking for the same amount of time during *S−* presentation resulted in a timeout interval of 7 s. In all other response cases, the intertrial interval was 3 s, and no water reward was delivered. All behavioral performance within a specified trial bin was calculated as a weighted average of *S*+ versus *S−* performance

$$\text{Performance}=\frac{\left(\text{Hit}/S+\right)+\left(\mathrm{CR}/S-\right)}{2}$$ (3)

in which *S*+ is the total number of rewarded trials, *S−* is the total number of unrewarded trials, Hit is the total number of rewarded trials in which a lick response was detected, and *CR* (correct rejection) is the total number of unrewarded trials in which no lick response was detected.
