# Supplementary material for: High-speed odor sensing using miniaturized electronic nose
Source: Sci Adv. 2024 Nov 6;10(45):eadp1764. doi: 10.1126/sciadv.adp1764 (PMC11540037; doi:10.1126/sciadv.adp1764)
Supplement: Supplementary file 1 — Figs. S1 to S3 [file sciadv.adp1764_sm.pdf]

Science Advances
AAAS

Supplementary Materials for

High-speed odor sensing using miniaturized electronic nose

Nik Dennler *et al.*

Corresponding author: Nik Dennler, dennler@proton.me; Michael Schmuker, m.schmuker@biomachinelearning.net

Sci. Adv. **10**, eadp1764 (2024)
DOI: 10.1126/sciadv.adp1764

This PDF file includes:

Figs. S1 to S3

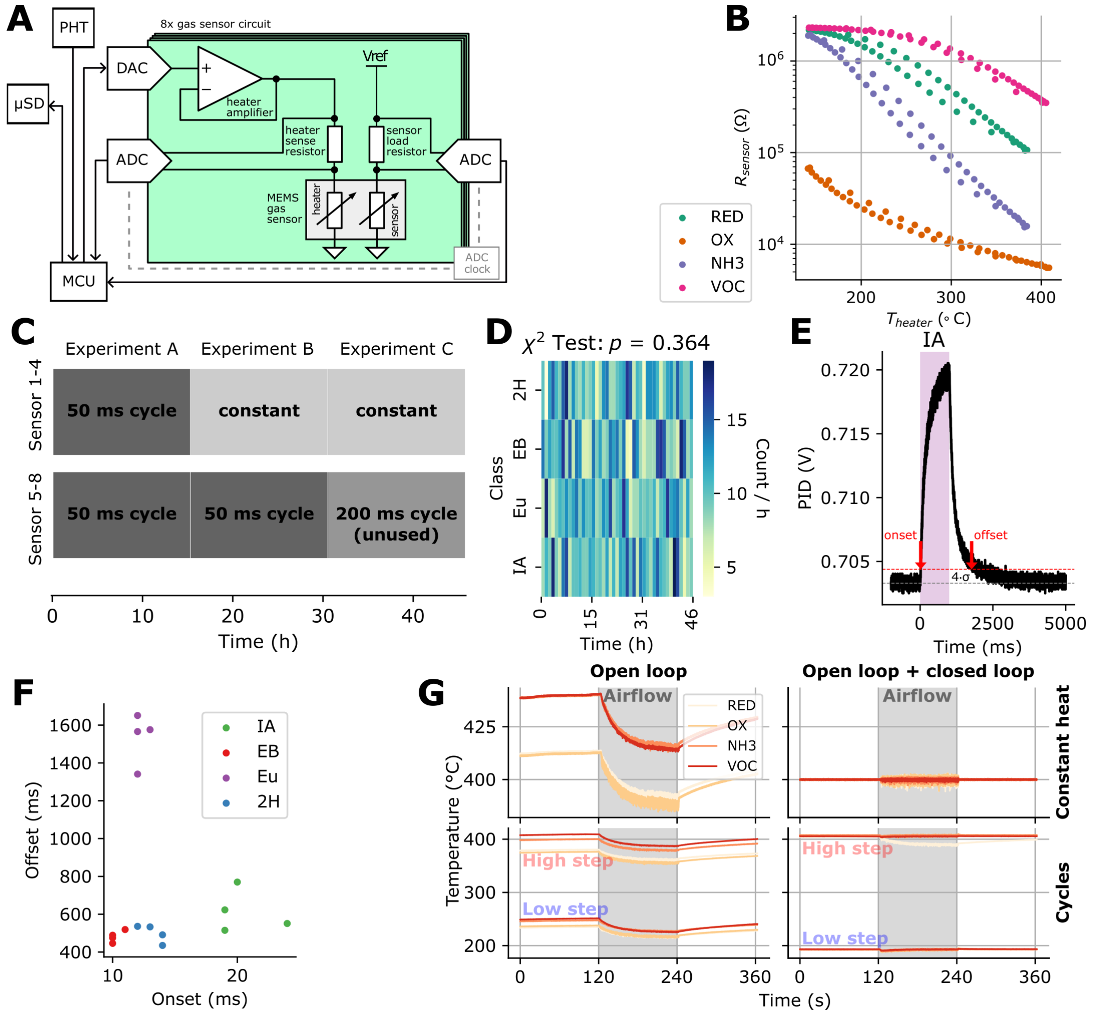

Figure S1: **Supplementary figure for experimental setup. A**, Electronic nose design, displaying how the microcontroller unit (MCU) sets and reads out the sensor heaters in a closed-loop, while reading out the analyte dependent sensor resistances. Further, the MCU connects to an environmental sensor (PHT) and a micro SD card. **B,** R-T curve of a $50\,\mathrm{ms}$ temperature cycle between $150\,°\mathrm{C}$ and $400\,°\mathrm{C}$ without external stimulus, displaying how the sensor response closely follows the hotplate temperature. **C,** Different sensor hotplate settings over time. For each experiments, all the stimuli were presented in randomised order. **D,** Heatmap depicting the distribution of odour presentations over a set of 1 hour time intervals. A χ^2 test was performed to assess the randomness of class distribution over time intervals, with the computed p-value indicated as 'p'. **E,** PID response to a $1\,\mathrm{s}$ isoamyl acetate pulse. Grey-dotted and red dotted lines denote mean of pre-stimulus baseline and 4 standard deviations threshold respectively. Where the response crosses the threshold upwards (downwards), the odour onset (offset) is registered. **F,** Extracted odour onsets (w.r.t. $t = 0\,\mathrm{ms}$) and offsets (w.r.t. $t = 1000\,\mathrm{ms}$) for $1000\,\mathrm{ms}$ pulses of different odours. **G,** Open-loop vs. open-loop + closed-loop control of sensor heaters, for constant heat and cycles.

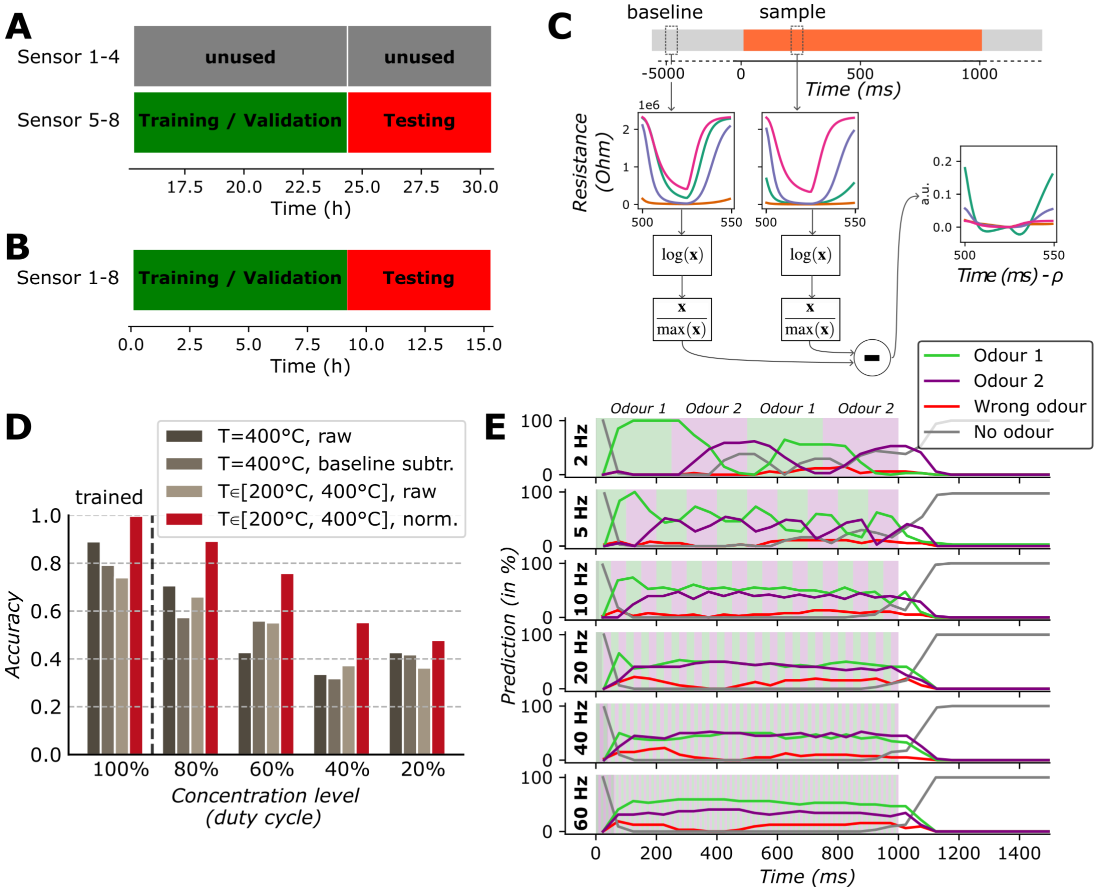

Figure S2: **Supplementary figure for fast odour c lassification. A,** Data splitting for robustness analysis of the rapid heater modulation data features (see Fig. 2E). **B,** Data splitting for evaluating the dynamic classification of millisecond odour pulses (see Fig. 3). **C,** Normalisation procedure for the heater modulation data feature. Time shifted by cycle phase ρ w.r.t. odour onset, for visual guidance only. **D,** Accuracy scores for a k-nearest neighbours (k-NN) classifier trained on $50\,\mathrm{ms}$ data features from $1000\,\mathrm{ms}$ odour pulses at full concentration, and tested on $50\,\mathrm{ms}$ features from $1000\,\mathrm{ms}$ odour pulses at different concentration levels (tuned by adjusting the duty cycle of the micro-valves). Features are compared for constant heater sensor readings (raw and baseline-normalised) and cycled heater sensor readings (raw and normalised, as described in c)) **E,** Classification correctness over time (evaluated via the true odour presence), for anti-correlated odour patterns of different switching frequencies.

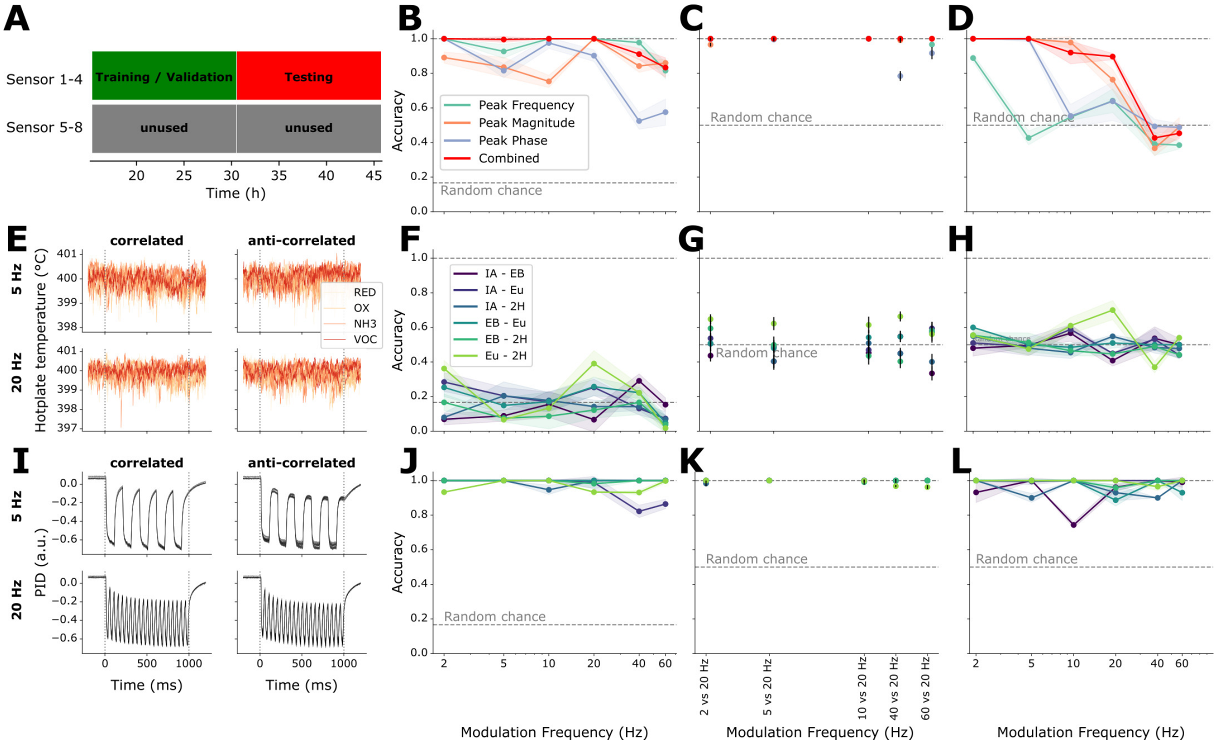

Figure S3: **Supplementary figure for temporal pattern discrimination. A,** Data splitting for evaluating the temporal pattern discrimination performance (see Fig. 4 B-D Validation accuracy plots for different extracted DFT-spectrogram peak features using the MOx gas sensor resistances. **B,** Modulation frequency classification, **C,** pairwise modulation frequency classification, and **D,** correlated vs anti-correlated modulation discrimination. **E,** MOx heater temperature values for different odour modulations. Here shown are data for the odour pair IA (isoamyl acetate)- EB (ethyl butyrate), 5 trials each for 5 Hz correlated, 5 Hz anti-correlated, 20 Hz Hz correlated and 20 Hz anti-correlated respectively. **F-H,** Test accuracy plots for different odour pair modulations, using the MOx heater temperature values. **I,** Photoionisation Detector (PID) responses for different odour modulations (odour stimuli as in **E**). **J-L,**, Test accuracy plots for different odour pair modulations, using the Photoionisation Detector (PID) responses. For all the classification tasks, an ensemble of Random Forest Classifiers was used. The mean and error estimations arise from repeating training and testing with different random seeds.
